# Fear of hypoglycemia is linked to poorer glycemic control and reduced quality of life in adults with type 1 diabetes

**DOI:** 10.3389/fendo.2025.1563410

**Published:** 2025-05-16

**Authors:** Ángel Manuel Mesa Díaz, Samuel Belmonte Lomas, Pablo Rodríguez de Vera Gómez, María Victoria Llanes González, Carmen Mateo Rodríguez, Lucía Hidalgo Sotelo, María Asunción Martínez Brocca

**Affiliations:** Endocrinology and Nutrition Department, Virgen Macarena University Hospital, Seville, Spain

**Keywords:** type 1 diabetes, fear of hypoglycemia, quality of life, flash glucose monitoring, glycemic control, hypoglycemia unawareness, ambulatory glucose profile, FH15 questionnaire

## Abstract

**Aim:**

Analyze the influence of Fear of Hypoglycemia (FoH) on quality of life and glycemic control in adults with Type 1 Diabetes Mellitus (T1D) who use Flash Glucose Monitoring (FGM)

**Methods:**

A cross-sectional study was conducted with 173 adults with T1D. FoH was assessed using the FH15 questionnaire, and quality of life was evaluated using the Spanish Diabetes Quality of Life questionnaire (EsDQOL). Glycemic control was analyzed through FGM-derived metrics (Ambulatory Glucose Profile) and HbA1c. Multivariate multiple linear regression models were developed to analyze the adjusted impact of FoH on quality of life and time in hyperglycemia.

**Results:**

FoH was present in 42% of participants (FH15 ≥28). Patients with FoH exhibited significantly higher EsDQOL scores, reflecting poorer quality of life, and higher HbA1c levels (7.41% vs. 7.08%, p=0.012). FGM metrics revealed higher mean glucose, glucose management indicator (GMI) (p=0.008), and time in hyperglycemia >250 mg/dL (p=0.035) in the FoH group, with lower time in range 70–180 mg/dl (p=0.035). Hypoglycemia unawareness was more frequent in the FoH group (25.4% vs. 6.5%, p=0.011). The FH15 score functioned as an independent predictor of quality of life (β = 1.98 [1.58; 2.37]) and time in hyperglycemia (β = 0.39 [0.17; 0.61]) in the multiple linear regression models.

**Conclusion:**

FoH is significantly associated with poor quality of life and worsened glycemic control in T1D patients, underscoring the need for systematic assessment and individualized interventions. FGM metrics, particularly time in hyperglycemia, may serve as valuable predictors of FoH. Comprehensive management strategies addressing both metabolic and psychological factors are essential for improving patient outcomes.

## 1 Introduction

Type 1 Diabetes Mellitus (T1D) is a chronic and complex disease that significantly impacts patients’ quality of life. In the clinical management of individuals with T1D, addressing psychosocial factors and quality of life issues has been proposed as a fundamental goal (1, 2).

Hypoglycemia is one of the most common complications in patients with diabetes undergoing intensive insulin therapy (3). It is defined as a blood glucose level below 70 mg/dL and can be classified into three levels: Level 1 hypoglycemia (blood glucose <70 mg/dL but ≥54 mg/dL), Level 2 (blood glucose <54 mg/dL), and Level 3 (severe events requiring third-party assistance to restore normal blood glucose levels, regardless of the glucose value) (4). Hypoglycemia can cause significant discomfort due to the occurrence of adrenergic symptoms (sweating, tachycardia, palpitations, etc.) and neuroglycopenic symptoms (behavioral alterations, instability, or hypoglycemic coma) (5). Recurrent hypoglycemia or severe events that interfere with daily activities may contribute to psychological distress, including unhappiness and anxiety-depressive spectrum disorders, leading to a reduction in quality of life and the development of avoidance behaviors that hinder optimal glycemic control (6, 7). This results in the phenomenon of FoH, defined as a generalized anxiety disorder characterized by a disproportionate anticipatory response to a perceived threat (initially real) experienced with significant distress and insecurity by the patient (7).

Interstitial glucose monitoring systems, both continuous (CGM) and intermittent or flash (FGM), are increasingly being adopted by patients with diabetes and have shown beneficial effects on glycemic control and quality of life (2, 8). While several factors associated with FoH (e.g., female gender, educational level, history of severe hypoglycemia) have been proposed, the detailed evaluation of its association with glycemic control or quality of life parameters remains unclear.

This study aims to investigate the influence of FoH on the quality of life and glycemic control of adult patients with T1D. This novel approach seeks to provide information to prioritize individualized therapeutic goals, improving metabolic control and patient well-being in those with FoH.

## 2 Materials and methods

### 2.1 Study design

A cross-sectional observational study was conducted in the Endocrinology and Nutrition Department of Virgen Macarena University Hospital (Seville, Spain) from January 1, 2023, to December 31, 2023. Participants were systematically and consecutively selected from the department’s internal patient registry, applying the following inclusion criteria: adults aged 18 years or older, confirmed diagnosis of T1D [ADA 2024 criteria (9)], at least one year of evolution since diagnosis of T1D, treatment with multiple daily insulin injections (MDI) or sensor-augmented insulin pump systems (open-loop).

Exclusion criteria included patients unable to understand, speak, or write in Spanish, those with severe cognitive impairments, minors, individuals with diagnoses other than T1D, users of hybrid closed-loop insulin delivery systems (HCL), and patients with sensor data capture rates <70%.

### 2.2 Variables

The primary variable was the presence of FoH, evaluated using the FH15 test (Fear of Hypoglycemia 15). This validated test for the Spanish population specifically assesses fear of hypoglycemia and demonstrates robust reliability parameters (10). It contains 15 predefined questions scored using a 5-point Likert scale, yielding a total score ranging from 15 to 75. A score of 28 or higher indicates the presence of pathological FoH (11). This questionnaire has been validated in other languages, such as Chinese and Italian (10, 12, 13), and has been used in recent studies by our group (14).

Perceived quality of life was evaluated using the Spanish version of the Diabetes Quality of Life questionnaire (EsDQOL), which includes 43 items divided into four subscales: impact of diabetes on daily life, diabetes-related concerns, satisfaction with the disease, and social concerns. Lower scores indicate better perceived quality of life (15).

Glycemic control was assessed using glucometric reports provided by the FGM systems (Ambulatory Glucose Profile, AGP), covering the 14 days preceding the completion of the questionnaires. All participants used the “FreeStyle Libre 2” system (Abbott Laboratories) for FGM.

The presence of hypoglycemia unawareness was evaluated using the Clarke test. This questionnaire detects hypoglycemia unawareness in individuals with diabetes on insulin therapy. It comprises eight items that assess symptoms perceived during hypoglycemic events, the frequency of such events, and the glucose thresholds at which symptoms occur. Responses are categorized as normal (A) or abnormal (R), with four or more abnormal responses indicating hypoglycemia unawareness (3).

Additional variables included sociodemographic and clinical factors (sex, age, age at disease onset, years of diabetes duration, educational level, cardiovascular risk factors, and presence of chronic complications) and HbA1c levels as an additional measure of glycemic control.

### 2.3 Statistical analysis

A minimum sample size of 170 participants was estimated for the study, assuming a minimum difference of six points in the primary variable (based on a prior study by our group (14)), with 80% power and a 95% confidence level.

Descriptive results were presented as mean and standard deviation (SD) or median and interquartile range (IQR), depending on the distribution assessed using the Shapiro-Wilk test. Parametric or non-parametric tests were used as appropriate.

The association between quantitative variables was analyzed using correlation analysis (Pearson’s correlation). For group comparisons, the chi-square test and Fisher’s exact test were used for dichotomous qualitative variables, and the Student’s t-test or Mann-Whitney U test for quantitative variables.

To evaluate the effect of FH on quality of life and glycemic control, two multivariable models were developed, with the EsDQOL score and the percentage of time in hyperglycemia >180 mg/dl as the dependent variables, respectively. Independent variables were selected using an automated stepwise method among those with p<0.15. Assumptions for multiple linear regression (linearity, normality and independence of residuals, homoscedasticity and multicollinearity) were verified.

Inferential statistical analyses were performed using a two-tailed design, with p<0.05 considered statistically significant. The software used was SPSS v.26.

### 2.4 Ethical aspects

This study was conducted following the ethical principles outlined in the Declaration of Helsinki and was approved by the Research Ethics Committee of Virgen Macarena and Virgen del Rocío University Hospitals (code TGH-IPCD-2023).

## 3 Results

### 3.1 Patients

A total of 173 individuals were included in the study. The mean age was 42.1 years (SD: 11.7), with 48.6% (n=84) being women. The mean duration of T1D was 22.4 years (SD: 12.7). The mean HbA1c level was 7.3% (SD: 0.8), with 17.6% (n=30) of participants having values >8%. A total of 16.2% (n=28) were users of sensor-augmented insulin pumps (Medtronic 640G, open-loop with FGM). These patients exhibited better glycemic control compared to the MDI group, in terms of HbA1c (6.95 vs. 7.34, p = 0.014) and time in hyperglycemia ≥250 mg/dL (6.12% vs. 11.74%, p = 0.001), with no differences in the psychosocial questionnaire scores used in the study (**Supplementary Material**). The main characteristics, glycemic control variables, and questionnaire scores are summarized in **Table 1**.

**Table 1:** Description of the sample and differences between the presence or absence of MH.

Study variables	TOTAL (n=173)	FH15<28 (n=69)	FH15≥28 (n=104)	Differences^2^	p-value
Age (years)^1^	42.1 (11.77)	40.88 (11.77)	42. 98(11.75)	-2.09 [-5.70; 1.50]	0.252
Sex					
Male Female	89 (51.40%) 84 (48.60%)	43 (62.30%) 26 (37.70%)	46 (44.20%) 58 (55.80%)	2.08 [1.12; 3.88]	0.02
Years with diabetes	22.4 (12.70)	20.37 (13,32)	23.76 (12.12)	-3.38 [-7.36; 0.58]	0.094
Age at debut	21.43 (12.40)	20.54 (13.08)	22.02 (12.00)	-1.47 [-5.29; 2.33]	0.445
Insulin treatment					
Multiple doses CSII amplified with MFG	145 (83.80%) 28 (16.20%)	59 (85.50%) 10 (14.50%)	86 (82.70%) 18 (17.30%)	1.24 [0.53; 2.86]	0.672
Microvascular complications					
Retinopathy Nephropathy Neuropathy	32 (18.50%) 17 (9.80 %) 6 (3.50%)	9 (13%) 4 (5.80%) 1 (1.40%)	23 (22.10%) 13 (12.5%) 5 (4.80%)	1.89 [0.81; 4.38] 2.32 [0.72; 7.44] 3.32 [0.72; 7.44]	0.132 0.147 0.237
Macrovascular complications					
Stroke Ischemic heart disease Peripheral artery disease	3 (1.70%) 4 (2.30 %) 5 (2.90%)	1 (1.40%) 0 (0%) 1 (1.40%)	2 (1.90%) 4 (3.80%) 4 (3.80%)	1.33 [0.12; 14.99] 0.59 [0.52; 0.67] 2.72 [0.29; 24.86]	0.815 0.099 0.357
Glycemic control					
Time with active sensor (%)	93.78 (10.71)	94.06 (9.11)	93.6 (11.70)	0.46 [-2.90; 3.83]	0.786
HbA1c (%)	7.3 (0.88)	7.06 (0.74)	7.41 (0.94)	-0.34 [-0.61; -0.07]	0.012
HbA1c categories					
<7% 7-8% >8%	64 (37.60%) 76 (44.70%) 30 (17.60%)	35 (51.50%) 27 (39.70%) 6 (8.80%)	29 (28.40%) 49 (48%) 24 (23.50%)	*	0.003
GMI	7.2 (0,74)	7.01 (0.59)	7.32 (0.79)	-0.31 [-0.53; -0.08]	0.008
Average blood glucose (mg/dl)	161.74 (38)	153.33 (27.96)	167.34 (42.64)	-14.01 [-25.77; -2.24]	0.020
% TIR (70-180 mg/dl)	61.47 (17.39)	64.95 (15.20)	59.14 (18.41)	5.81 [0.41; 11.21]	0.035
% TAR I (181-249 mg/dl)	23.3 (9.73)	21.77 (9.99)	24.31 (9.47)	-2.54 [-5.57; 0.49]	0.101
% TAR II (≥250 mg/dl)	10.8 (11.51)	8.58 (7.72)	12.42 (13.28)	-3.84 [-7.42; -0.27]	0.035
% TBR I (55-69 mg/dl)	3.68 (3.34)	3.86 (3.34)	3.57 (3.35)	0.29 [-0.75; 1.35]	0.575
% TBR II (≤54mg/dl)	0.69 (1.72)	0.89 (2.47)	0.56 (0.92)	0.34 [-0.20; 0.88]	0.216
Number of hypoglycemia events (<70 mg/dl)	10.62 (9.83)	11.09 (10.13)	10.31 (9.66)	0.77 [-2.31; 3.87]	0.620
Mean duration of hypoglycemia events (minutes)	82.85 (38.46)	89.62 (40.71)	78.34 (36.39)	11.27 [-0.70; 23.25]	0.065
Questionnaire scores					
ESDQL	92.43 (23.78)	75.85 (17.08)	104.08 (20.75)	-28.23 [-34.72; -21.74]	1.40E-14
ESDQL (satisfaction)	33.61 (9.87)	28.15 (7.03)	37.48 (9.79)	-28.23 [-12.22; -6.43]	2.47E-9
ESDQL (impact)	35.47 (10.85)	29.02 (6.93)	40.02 (10.83)	-11.00 [-14.14; -7.86]	1.33E-10
ESDQL (social/vocational)	13.53 (5.67)	10.89 (3.80)	15.39 (6.03)	-4.50 [-6.23; -2.77]	8.10E-7
ESDQL (diabetes)	9.73 (2.82)	7.98 (2.20)	10.95 (2.57)	-2.97 [-3.77; -2.17]	1.42E-11
Presence of inadvertent hypoglycemia (Clarke Test, R≥4)	18 (17.10%)	3 (6.50%)	15 (25.40%)	4.89 [1.32; 18.1]	0.011
R number in Test Clarke	1.87 (1.55)	1.39 (1.22)	2.24 (1.69)	-0.85 [-1.43; -0.26]	0.005

CSII, Continuous subcutaneous infusion of insulin; MFG, Monitoring flash of glucose; GMI, glucose management index; HbA1c, glycosylated hemoglobin; ESDQOL, Diabetes Quality of Life in Spanish; FH, Fear of Hypoglycemia; TIR, time in range; TAR, time above range (I: 181-249 mg/dl; II: ≥250 mg/dl); TBR, time below range (I: 55-69 mg/dl; II: ≤54 mg/dl).

^1^Data expressed as mean and SD (standard deviation) in quantitative variables and n (%) in qualitative variables.

^2^The differences have been expressed as mean differences in quantitative variables, and as Odds ratios in qualitative variables. In both cases, 95% confidence intervals have been included.

### 3.2 Fear of hypoglycemia, quality of life, and hypoglycemia unawareness

Analysis of quality-of-life outcomes based on the presence or absence of FoH showed higher EsDQOL scores (indicative of poorer perceived quality of life) in the group with FH15 scores ≥28 points (presence of FoH) (**Table 1**). This was statistically significant for both the total score (mean difference: 92.43 vs 75.85 points, p=1.40E-14) and individual subscales (satisfaction, impact, social/vocational, and diabetes-related concerns) (**Table 1**).

**Figure 1:**
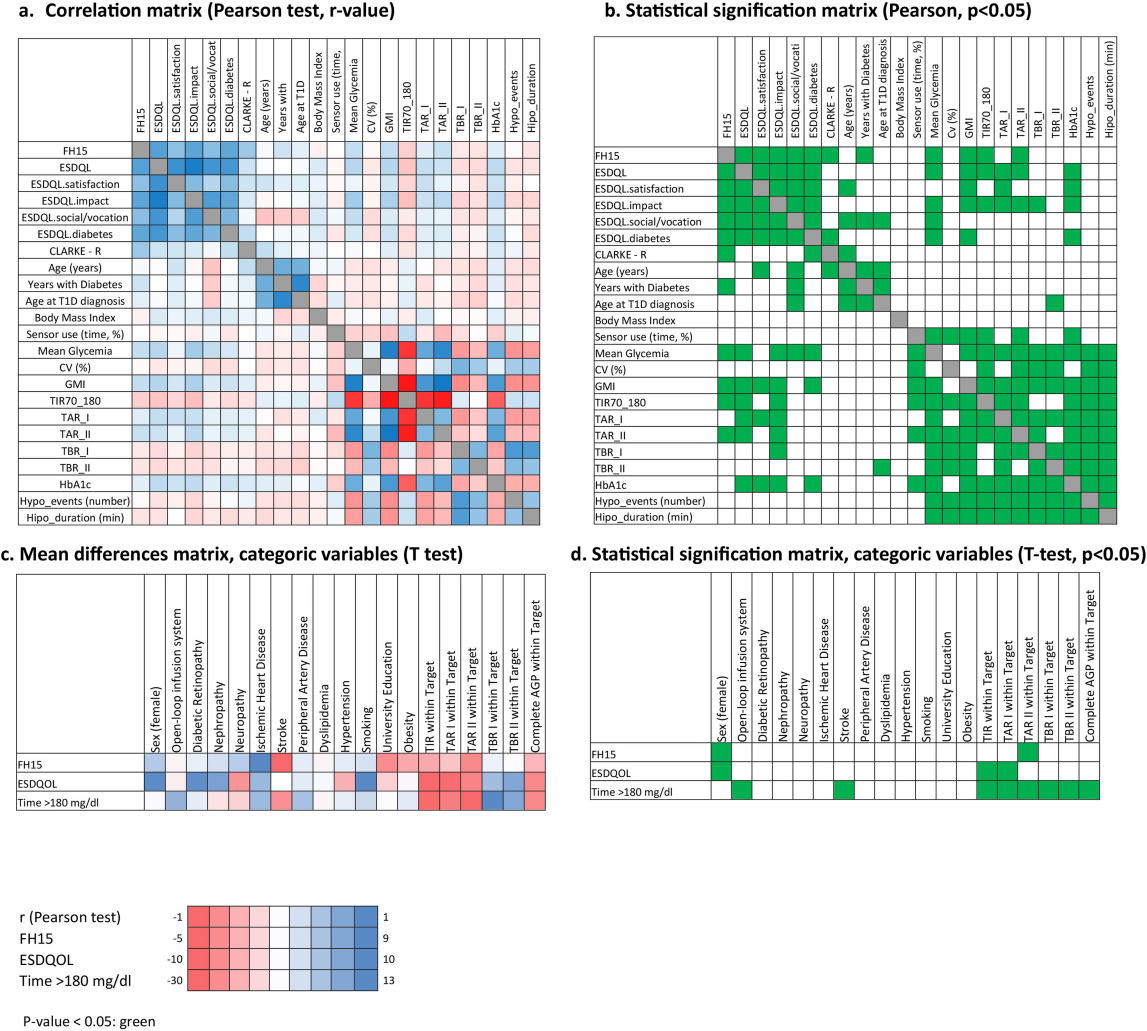
Univariate analysis of study variables. The correlation between quantitative variables was explored using Pearson’s test **(a, b)**, and mean differences between categorical variables in fear of hypoglycemia (FH15 score), quality of life (EsDQOL score), and time in hyperglycemia >180 mg/dl were assessed using the T-Student test **(c, d)**.

Regarding the association with other clinical variables, the proportion of women was higher in the FoH group compared to the non-FoH group (55.8% vs. 37.3%, p=0.02) (**Table 1**). Additionally, a directly proportional and statistically significant correlation was found between years with T1D and FoH (r=0.2; p=0.013) (**Figure 1**).

**Figure 2:**
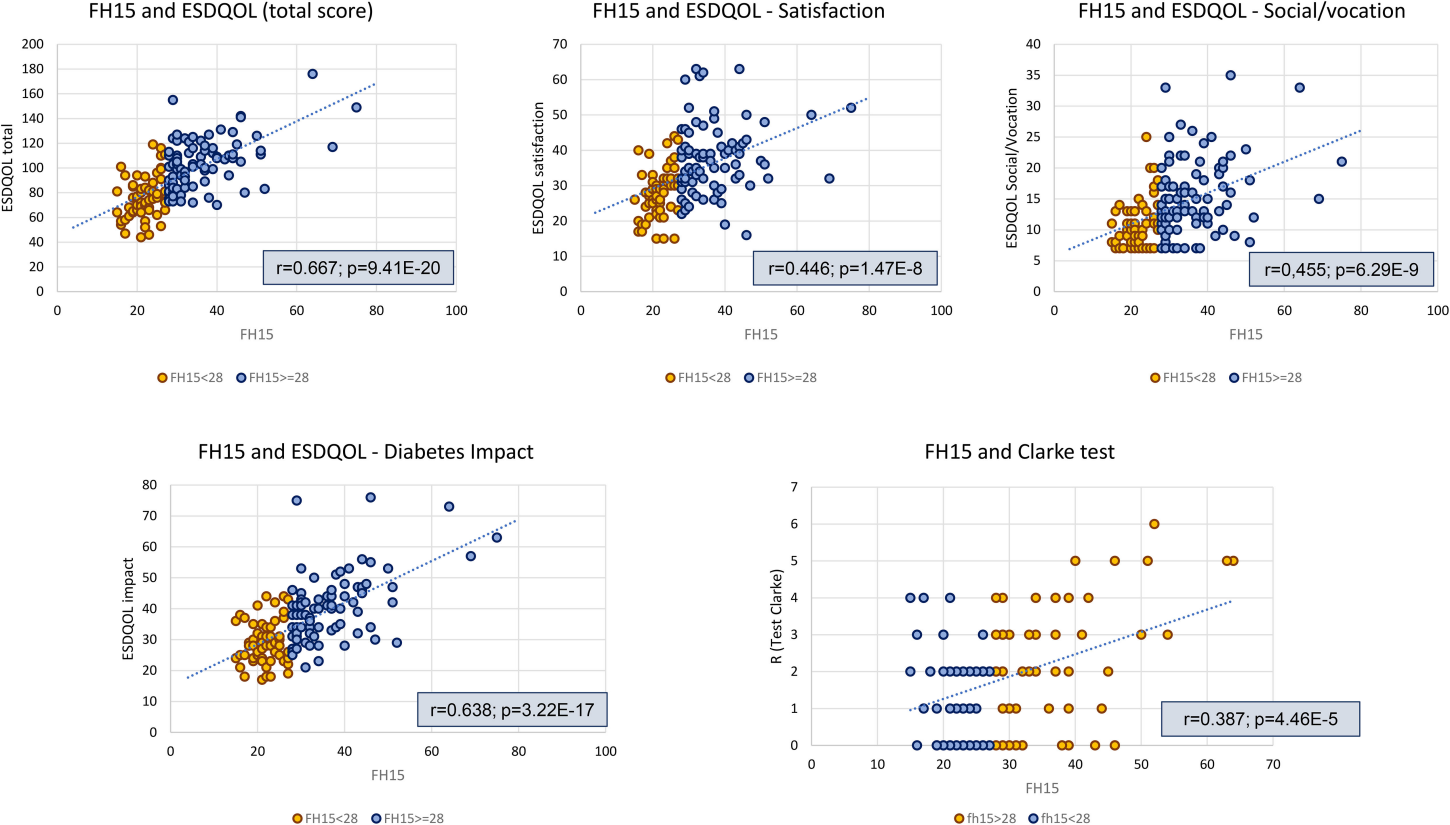
Analysis of linear correlation between FH15 scores and ESDQOL and Clarke questionnaire scores. ESDQOL, Diabetes Quality of Life in Spanish; FH, Fear of Hypoglycemia.

On the other hand, significant correlations were observed between FH15 and the total EsDQOL score (r=0.667; p=9.41E-20). This pattern was also confirmed in the correlation analysis between FH15 and the EsDQOL subscales (**Figure 2**), indicating that higher FoH values were associated with poorer perceived quality of life.

Regarding hypoglycemia unawareness, it was more frequent in the FoH group (participants with ≥4 abnormal responses: 25.4% in the FoH group vs. 6.5% in the non-FoH group, p=0.011) (**Table 1**). In this regard, the correlation analysis revealed a direct and statistically significant association between FH15 scores and the Clarke test (r = 0.4, p = 4.5E-5; **Figure 2**). When dividing the sample based on the presence or absence of hypoglycemia unawareness, significant differences in FH15 scores were observed between the groups, with higher MoH values in the group with Clarke ≥4 (38.1 (SD 13.8) points vs. 28.3 (SD 8.1), respectively; p = 0.009). The percentage of individuals with FH15 ≥28 (indicative of MH) was 6.5% in the group without hypoglycemia unawareness compared to 25.4% in the group with this complication (Odds ratio 4.9, 95% CI [1.3; 18.1], p = 0.11).

### 3.3 Fear of hypoglycemia and glycemic control

The mean HbA1c level was higher in the FoH group compared to the non-FoH group (7.41% vs. 7.08%; mean difference: -0.34 [-0.61; -0.07], p=0.012), with a higher proportion of participants exhibiting poor glycemic control (HbA1c >8%: 23.5% vs. 8.8%, p=0.003). Patients achieving glycemic targets (HbA1c <7%) accounted for 51.5% in the non-FoH group compared to 28.4% in the FoH group (p=0.003) (**Table 1**).

**Figure 3:**
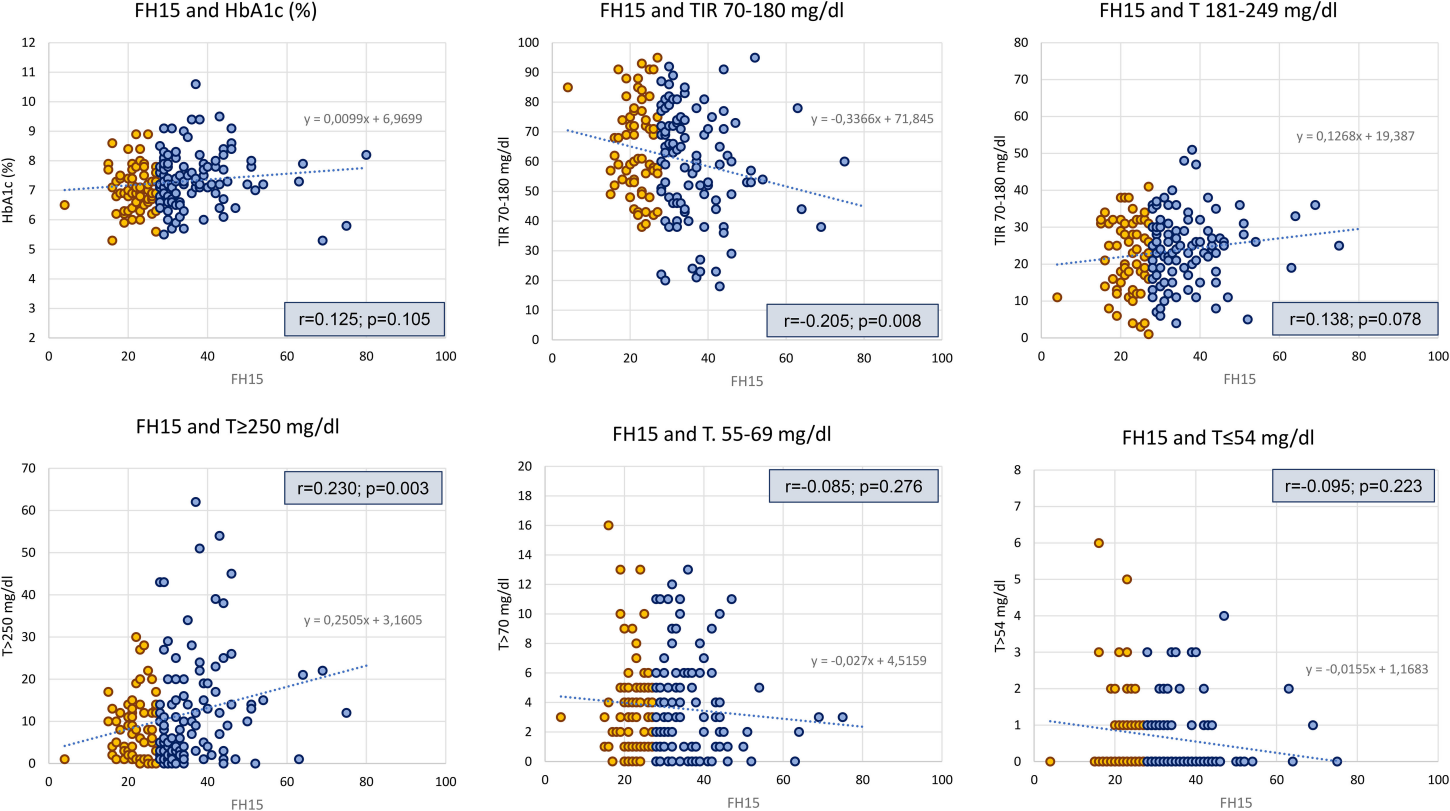
Analysis of linear correlation between FH15 scores and glycemic control variables. HbA1c, hemoglobin glycosilade; T, Time; TIR, Time in range; FH, Fear of Hypoglycemia.

AGP data analysis showed that patients with FoH had higher mean glucose levels (p=0.02), GMI (p=0.008), and time in hyperglycemia >250 mg/dL (p=0.035), along with lower time in range 70–180 mg/dL (TIR; p=0.035) compared to participants without FoH. No significant differences were observed between groups in terms of hypoglycemia. These findings were confirmed in univariate correlation analyses (Pearson test for FH15 and TIR: r=0.205, p-value: 0.008; FH15 and T≥250mg/dl: r=0.23, p-value: 0.003) (**Figures 1**, **3**).

The percentage of patients meeting all AGP target parameters (combining TIR, time >180 mg/dL, time >250 mg/dL, time <70 mg/dL, and time <54 mg/dL) according to the Battelino et al. consensus (16) was 18.2% in the FoH group and 16.7% in the non-FoH group (p=0.838).

### 3.4 Predictors of fear of hypoglycemia: multivariate model

To evaluate the influence of FoH on quality of life and hyperglycemia, two multiple linear regression models were developed (**Table 2**).

**Table 2:** Predictive factors of quality of life and time in hyperglycemia.

A. Multivariable linear regression model: Predictive factors of Quality of Life (ESDQOL questionnaire)		
	Coefficient [95% Confidence Interval]	p-value
FH15 score Years with T1D HbA1c Sex, female Hypoglycemia unawareness	1.98 [1.58; 2.37] -0.42 [-0.66; -0.17] 4.94 [1.18; 8.71] 5.93 [-0.74; 12.59] -6.19 [-15.29; 2.91]	6.06E-16 0.001 0.011 0.081 0.18
R2: 0.625, p= 8.59E-17. Dependent variable: ESDQOL questionnaire score.		
B. Multivariable linear regression model: Predictive factors of Time in Hyperglycemia (>180 mg/dl)^1^		
	Coefficient [95% Confidence Interval]	p-value
FH15 score Coefficient of Variation Time in hypoglycemia <70 mg/dl HbA1c Years with T1D	0.39 [0.17; 0.61] 0.64 [0.16; 0.29] -1.22 [-1.8; -0.64] 10.8 [8.25; 13.36] -0.23 [-0.38; -0.07]	0.001 5.71E-5 5.77E-5 4.89E-14 0.006
R2: 0.566, p= 1,64E-24. Dependient variable, Time in Hyperglycemia >180 mg/dl.		

Multivariate models. ^1^This variable has been calculated by adding the hyperglycemia values between 180-249 mg/dl and >250 mg/dl.

The verification of assumptions for multiple linear regression in both models is detailed in the **Supplementary Material**.

HbA1c, hemoglobin glycosilade; ESDQOL, Diabetes Quality of Life in Spanish; FH, Fear of Hypoglycemia.

In the first model, with EsDQOL score as the dependent variable, the FH15 score showed a strong influence (β = 1.98 [1.58; 2.37], p = 6.06E-16), indicating that for each one-point increase in FH15, the EsDQOL score increased by 1.98 points. Other included variables were years with T1D, HbA1c level, sex, and the presence of hypoglycemia unawareness, resulting in a coefficient of determination R² = 0.625, meaning that 62.5% of the variability in EsDQOL was explained by the model.

In the second model, with time in hyperglycemia >180 mg/dl (TAR>180) as the dependent variable, the FH15 score has a significative coefficient (β = 0.39 [0.17; 0.61], p = 0.001), indicating that for each one-point increase in FH15, the TAR>180 mg/dl increased by 0.39 percent. Additional included variables were coefficient of variation (CV), time below range (TBR <70 mg/dl), HbA1c level, and years with diabetes, leading to an R² = 0.566, suggesting that 56.6% of the variability in TAR was explained by the model.

The verification of multiple linear regression assumptions for both models can be found in the **Supplementary Material**.

## 4 Discussion

In this study, we evaluated the relationship and effect of FoH on glycemic control and perceived quality of life in individuals with T1D. One of the novelties of this work lies in the specific assessment of the relationship between fear of hypoglycemia and its association with glycemic control using interstitial continuous glucose monitoring metrics. This approach aims to identify at-risk patients and optimize therapeutic decision-making.

Our results demonstrate a strong correlation between FoH scores (FH15 questionnaire) and quality of life (EsDQOL questionnaire), indicating that patients with higher levels of FoH report poorer perceived quality of life. This association was confirmed in the multiple linear regression model, where the FH15 score functioned as a predictive factor for EsDQOL, along with a shorter duration of T1D, a higher HbA1c level, and the presence of unawareness of hypoglycemia. Similarly, literature reviews have concluded that identifying and addressing FoH is essential to implementing measures aimed at improving quality of life in individuals with T1D and reducing the anxiety and distress associated with the condition (7). The psychological impact of the disease, and FoH in particular, can significantly limit the daily lives of individuals with T1D, affecting their daily activities, personal relationships, and work productivity, with a multifaceted impact on patients’ lives (17). Carrie Fidler et al. (18) conducted a narrative review analyzing how FoH affects the quality of life of patients with diabetes on insulin therapy and the costs associated with this phenomenon. They concluded, in line with our findings, that FoH significantly impacts quality of life and has considerable economic implications, increasing direct healthcare costs and indirect costs due to lost productivity. It generates chronic anxiety, sleep disturbances, and compromises social and professional life while fostering treatment changes that negatively affect metabolic control. This suboptimal management contributes to long-term higher costs for healthcare systems and society (19).

Our study also highlights the association between FoH and glycemic control, demonstrating that individuals with higher FoH levels exhibit worse glycemic control, including higher HbA1c, mean glucose, and time in hyperglycemia, as shown by AGP data. These findings align with prior reports on FoH and CGM (14), where undertreatment due to fear of hypoglycemia is suggested as a likely primary cause of this deterioration. Other studies (20) have concluded that the negative influence on metabolic control in individuals with T1D involves increased glycemic variability and impaired self-care behaviors, leading to inappropriate dietary adjustments, such as excessive calorie intake, and limited physical activity. Additionally, FoH is linked to excessive or inadequate responses to hypoglycemic episodes, contributing to extreme glucose fluctuations. Our study confirms these associations, with particular interest in the multiple linear regression analysis results, which show that higher FH15 scores, a high coefficient of variation, and reduced time in hypoglycemia are predictive factors for time >180 mg/dL. It is possible that the adoption of avoidant behaviors in response to potential hypoglycemic episodes contributes to poorer glycemic control, primarily due to excessive time spent in hyperglycemia.

It is important to note that FoH is not limited to patients with poor metabolic control. In our sample, 28.3% of patients with FH15 scores ≥28 had HbA1c <7%, while only 23.5% had HbA1c >8%. This underscores the need for systematic assessment of FoH in all patients, regardless of their glycemic control levels (20, 21). Moreover, it would be interesting to design a specific study aimed at identifying factors associated with why some individuals with high FoH achieve good glycemic control while others do not. In our study, there were no differences in the percentage of hypoglycemic episodes between patients with and without FoH. This suggests that FoH is a psychopathological phenomenon likely related to past experiences (e.g., severe hypoglycemia (6, 22)) and predisposing personality traits rather than constant exposure to hypoglycemia (23).

Our findings, highlighting the association with both quality of life and glycemic control, emphasize the need for regular FoH evaluation in clinical practice and the development of effective strategies to address it. The implementation of FGM has been shown to significantly improve this phenomenon and, consequently, perceived quality of life, as demonstrated in previous studies (14). Additionally, the literature documents that using hybrid closed-loop systems could be an effective strategy to improve both glycemic control and FoH by reducing the incidence of hypoglycemia (24, 25). Cohen et al. (26) conducted a clinical trial evaluating whether closed-loop systems improved glycemic control and FoH compared to multiple daily insulin injections, concluding that both aspects improved significantly. Structured educational interventions and cognitive-behavioral therapies have also been reported to reduce this phenomenon (27).

Studies have shown that female sex confers a higher risk of anxiety/depressive disorders (28), also in relation to diabetes (29, 30), as supported by our study findings. The higher frequency of FoH in women may therefore be related to this greater prevalence of anxiety disorders (31). It would be valuable to further investigate the causes of poorer perceived quality of life among women using a qualitative research approach that incorporates patient expectations (PREMS) with a gender perspective (32).

Our results also indicate that individuals with longer diabetes duration exhibit higher levels of fear of hypoglycemia. While our literature review did not identify specific studies analyzing this finding, other studies suggest that FoH may be intensified by factors such as previous episodes of severe hypoglycemia or the frequency of hypoglycemia, which could indirectly relate to patients with longer disease duration and accumulated experiences (14, 33).

Another noteworthy finding in our study is the association between FoH and hypoglycemia unawareness, as evidenced by higher Clarke test scores in the FoH group, higher FH15 scores in the hypoglycemia unawareness group, and a statistically significant positive correlation between the scores of both questionnaires. Hypoglycemia unawareness refers to episodes lacking the neuroglycopenic symptoms that alert individuals to hypoglycemia (4). This increases the risk of severe episodes by hindering early detection of low glucose levels, causing greater concern and anxiety and leading to compensatory strategies that negatively impact diabetes management and quality of life (29).

This study has several strengths, including the use of CGM to predict the presence of FoH. This technology provides a valuable tool for evaluating glycemic control and identifying relevant patterns in clinical practice to detect FoH. We identified that time in hyperglycemia among individuals with T1D behaved as a predictive variable for FoH. To our knowledge, this is the first study to establish the association between FoH and time in hyperglycemia. This finding is relevant as it highlights potential implications for clinical practice and future research to study the impact of interventions aimed at reducing time in hyperglycemia and FoH.

We recognize certain limitations in our research. Patients using hybrid closed-loop (HCL) systems were excluded from the analysis, which may have introduced a selection bias. These devices have revolutionized the management of T1D, demonstrating significant improvements in glycemic control, TIR, and reduced hypoglycemia rates. Additionally, they have been shown to alleviate the emotional burden associated with diabetes, positively impacting quality of life and reducing distress related to the disease and FoH (34). Consequently, excluding this patient group may limit the generalizability of our findings. The rationale for their exclusion was to ensure a homogeneous sample representative of a specific population—adults with T1D using non-automated insulin administration methods—while also minimizing measurement bias due to the use of different interstitial glucose monitoring (CGM) systems.

Another limitation is the exclusion of patients using CGM for less than 70% of the time. This criterion was established to ensure sufficient validity of AGP data; however, it may introduce selection bias by favoring more adherent patients.

A relevant aspect identified in the literature is the potential influence of nocturnal hypoglycemia on FoH, a factor not considered in our study. Previous reviews have shown that of all the articles included, only one demonstrated a positive association between nocturnal hypoglycemia frequency and FoH (17, 23). This could be a promising line of future research, as the relationship between nocturnal hypoglycemia and FoH is critical for developing therapeutic strategies, particularly those aimed at reducing the emotional impact associated with hypoglycemia.

In conclusion, our findings highlight the significant association between the presence of fear of hypoglycemia and a decline in perceived quality of life and glycemic control in individuals with T1D. We recommend the systematic assessment of FoH in individuals with T1D, regardless of glycemic control, including those with HbA1c levels within the target range, as a means to improve psychological well-being. Our results reinforce the importance of an integrated approach that encompasses both glycemic control and psychological factors associated with fear of hypoglycemia, ultimately contributing to the overall well-being of individuals with T1D.

## Data Availability

The raw data supporting the conclusions of this article will be made available by the authors, without undue reservation.
